# Dobutamine Stress Echocardiography Safety in Chagas Disease Patients

**DOI:** 10.5935/abc.20170002

**Published:** 2017-02

**Authors:** Daniela do Carmo Rassi, Marcelo Luiz Campos Vieira, Rogerio Gomes Furtado, Fabio de Paula Turco, Luciano Henrique Melato, Viviane Tiemi Hotta, Colandy Godoy de Oliveira Nunes, Luiz Rassi Jr., Salvador Rassi

**Affiliations:** 1 Faculdade de Medicina da Universidade Federal de Goiás (UFG) - Brazil; 2 Centro de Diagnóstico por Imagem (CDI), Goiânia, GO - Brazil; 3 Hospital São Francisco de Assis; Goiânia, GO - Brazil; 4 Instituto do Coração (InCor) - Faculdade de Medicina da Universidade de São Paulo, São Paulo, SP - Brazil

**Keywords:** Chagas Disease, Echocardiography, Stress, Atropine, Trypanosoma cruzi / drug effects

## Abstract

**Background:**

A few decades ago, patients with Chagas disease were predominantly rural workers, with a low risk profile for obstructive coronary artery disease (CAD). As urbanization has increased, they became exposed to the same risk factors for CAD of uninfected individuals. Dobutamine stress echocardiography (DSE) has proven to be an important tool in CAD diagnosis. Despite being a potentially arrhythmogenic method, it is safe for coronary patients without Chagas disease. For Chagas disease patients, however, the indication of DSE in clinical practice is uncertain, because of the arrhythmogenic potential of that heart disease.

**Objectives:**

To assess DSE safety in Chagas disease patients with clinical suspicion of CAD, as well as the incidence of arrhythmias and adverse events during the exam.

**Methods:**

Retrospective analysis of a database of patients referred for DSE from May/2012 to February/2015. This study assessed 205 consecutive patients with Chagas disease suspected of having CAD. All of them had their serology for Chagas disease confirmed.

**Results:**

Their mean age was 64±10 years and most patients were females (65.4%). No patient had significant adverse events, such as acute myocardial infarction, ventricular fibrillation, asystole, stroke, cardiac rupture and death. Regarding arrhythmias, ventricular extrasystoles occurred in 48% of patients, and non-sustained ventricular tachycardia in 7.3%.

**Conclusion:**

DSE proved to be safe in this population of Chagas disease patients, in which no potentially life-threatening outcome was found.

## Introduction

Chagas disease continues to be a serious health problem, as well as an economic burden in most Latin-American countries. The World Health Organization has recently estimated that 18 million people are chronically infected with *Trypanosoma cruzi,* and approximately 200,000 new cases are diagnosed per year.^1^

A few decades ago, patients with Chagas disease were mainly rural workers, with low risk profile for coronary artery disease (CAD). As urbanization has increased since 1980, they became exposed to the same risk factors for CAD of uninfected individuals. Thus, the prevalence of CAD, as a cause of acute myocardial infarction, is expected to be similar in individuals with and without Chagas disease.^2^

The prevalence of CAD in patients with Chagas disease, however, is controversial.^3-7^ It is worth noting the inherent diagnostic difficulty concerning chest pain, which can be atypical or intense.^8,9^ Coronary angiography should only be indicated in special situations, such as typical angina and presence of classic CAD risk factors, or when large ischemic areas are seen on non-invasive tests.^9^

For 25 years, stress echocardiography has proven to be an important tool for the diagnosis of CAD. The dobutamine-atropine protocol [dobutamine stress echocardiography - DSE)] is safe and has accuracy similar to that of other non-invasive diagnostic methods, but higher specificity.^10^

Dobutamine is the most commonly used agent in most pharmacological stress tests.^11,12^ Severe ventricular arrhythmias can occur during the exam, but are rare, confirming, thus, the safety of using dobutamine for stress echocardiography.^13,14^

In clinical practice, however, the indication of DSE in chronic Chagas heart disease (CCHD) is controversial, because of the arrhythmogenic potential of the drug in an also arrhythmogenic heart disease. In the literature, there is no study aimed at specifically assessing the safety of DSE in a group of Chagas disease patients. Thus, this research, aimed at assessing the DSE safety for CAD diagnosis in that group of patients, is relevant.

## Methods

### Selection of patients and study site

This is a retrospective analysis of a database to raise a hypothesis. A population of consecutive patients with CCHD and suspected of having CAD was assessed. They were referred for DSE from May/2012 to February/2015 at two echocardiography centers, one of them outside a hospital.

Confirmation of serology for Chagas disease was required in all patients. Those who spontaneously presented with at least two positive serologies at the time of DSE were confirmed as having Chagas disease. Those who had no serology were invited, via telephone, to undergo the serological tests, and to provide written informed consent at the time of blood sample collection. They underwent at least two serological tests of different principles, which confirmed the existence of anti-*T. cruzi* antibodies. The following conventional serological tests were used: immunoenzymatic assay (ELISA); indirect immunofluorescence; and indirect hemagglutination assay. Patients refusing to undergo the tests were excluded from the analysis.

### Echocardiographic assessment, analysis of safety and arrhythmias

Before DSE, the patients were asked about previous cardiovascular diseases, including Chagas disease, heart procedures they had already undergone, and regularly used medications.

Echocardiography was performed with a HD-11 echocardiographer (Philips Ultrasound Systems, Andover, MA, USA), by an echocardiography professional from a group of four, equally trained, and in a standardized and uniform way, according to the ASE recommendations.^15^ That group of professionals has a large experience with stress echocardiography, each performing, on average, 200 exams/month. The exams were performed systematically in all participants, regardless of the serological confirmation for Chagas disease.

Initially, the patients underwent a baseline echocardiographic study, with linear measurement of the heart structures and valvar flows. Ejection fraction was assessed by using the Teichholz or Simpson method, depending on the extent of the segmental contractility alteration. When using the latter, sometimes the end-systolic diameter was not measured. After acquiring baseline standard images in the parasternal, longitudinal, transverse, 4- and 2-chamber apical views, intravenous dobutamine infusion began, at an initial dose of 5 *µ*g/kg/min, with increasing increments of 10, 20, 30 and 40 *µ*g/kg/min every 3 minutes. If the patient had no echocardiographic sign of myocardial ischemia and did not reach the minimum heart rate of 100 bpm in the stage of 20 *µ*g/kg/min, 0.25 mg/min of atropine was administered every 1 minute, up to the maximum cumulative dose of 2 mg. There was no standardization concerning monitoring time after the end of infusion, and the time necessary for heart rate to reach less than 100 beats per minute was respected.

The patients were kept under clinical, electrocardiographic and continuous blood pressure monitoring. The measures of blood pressure, heart rate and 12-lead electrocardiography were recorded at baseline, at the end of each stage and during recovery. The patients' symptoms were recorded either through direct questioning or direct patient's complaint at any time.

The DSE was effective when one of the following objectives was met: at least 85% of maximum heart rate predicted for age, calculated with the Karvonen equation (maximum heart rate = 220 - age);^16^ echocardiographic signs of ischemia (new changes in left ventricular contractility); or end of the infusion protocol.

The submaximal criteria for test interruption, considered non-diagnostic were: unbearable symptoms; limiting side effects, such as arterial hypertension (systolic blood pressure > 230 mm Hg or diastolic blood pressure > 120 mm Hg); relative or absolute hypotension (systolic blood pressure drop > 30 mm Hg at rest, or systolic blood pressure < 80 mm Hg); supraventricular arrhythmias (sustained supraventricular tachycardia and atrial fibrillation); and ventricular arrhythmias (non-sustained and sustained ventricular tachycardia).^17^

The safety criteria for exam interruption were established as life-threatening complications, defined in the meta-analysis by Geleijnse et al.^18^ as cardiac rupture, acute myocardial infarction, stroke, asystole, ventricular fibrillation, and sustained ventricular tachycardia.

The cardiac arrhythmias observed during the exam were defined as follows: supraventricular tachycardia, presence of well-defined, regular and similar narrow QRS complexes (<120 ms), in the absence of conduction disorder; atrial fibrillation, absence of P wave associated with irregular rhythm, narrow QRS complexes (<120 ms), in the absence of conduction disorder; frequent ventricular extrasystoles, presence of premature ventricular complexes with more than 6 complexes per minute; ventricular bigeminism, presence of ventricular extrasystoles alternating with normal QRS complexes; non-sustained ventricular tachycardia, presence of more than 3 premature complex ventricular beats, lasting less than 30 seconds and with heart rate greater than 100 beats per minute; and sustained ventricular tachycardia, presence of more than 3 premature complex ventricular beats, lasting more than 30 seconds and with heart rate greater than 100 beats per minute.^19^

The left ventricle was divided into 17 myocardial segments, according to the ASE recommendations.^15^ The qualitative analysis of segmental myocardial contractility was based on visual assessment of myocardial thickening and wall motility graded into a segmental contractility index, each segment being scored as follows: 1 - normal; 2 - hypokinesia; 3 - akinesia; and 4 - dyskinesia. The normal value of that index is 1 (17 points/17 segments). Any value greater than 1 was considered abnormal segmental contractility index. Segmental myocardial contractility was positive for ischemia in the presence of altered segmental myocardial contractility in at least one left ventricular segment during pharmacological stress.^13,15^

### Statistical analysis

Non-probability convenience sampling was chosen and comprised patients with Chagas disease, suspected of having CAD, referred for DSE in the predetermined study period. The sample size was limited to the study's operational capacity.

Multivariate analysis was conducted using binary multiple logistic regression to identify covariables associated with the occurrence of binary outcome. When indicated, given the reduced number of binary outcome events, the use of penalized maximum likelihood ratio test was considered.

Multiple regression models were determined with the simultaneous introduction (*full model*) of the variables with p<0.05 in univariate regression analysis and that showed neither multicollinearity nor percentage loss greater than 10%.

Categorical variables were described as counts and percentages. Quantitative variables of normal and asymmetric distribution were described as mean ± standard deviation or median (interquartile range), respectively.

Normality was assessed via visual inspection of histograms. The R software (R Foundation, Vienna, Austria) was used for statistical analysis. All probabilities of significance presented are bilateral, and values smaller than 0.05 were considered statistically significant.

## Results

The general population referred for DSE underwent 23,935 exams. Of that sample, 415 patients claiming to have Chagas disease were selected. Of those, 210 patients whose serology was not confirmed were excluded, resulting in a final group of 205 patients to be assessed.

The mean age of the 205 patients analyzed was 64±10 years, and most of them (65.4%) were of the female sex. Regarding pharmacological treatment, the most used drugs were angiotensin II receptor blockers (35,1%) and amiodarone (29.3%). Of the reported risk factors for CAD, dyslipidemia was the most frequent (33.2%). Regarding the presence of previous coronary event, 6.3% reported myocardial infarction, and 5.9% surgical or percutaneous myocardial revascularization. Table 1 shows the clinical characteristics of the group and pharmacological treatment, and Table 2, the risk factors for CAD.

**Table 1:** Clinical characteristics of the total sample

Characteristic	
**Age (years) **	64±10 (Mean ± SD)
**Sex **	n= 205
Female	65.4%
**Drug treatment **	n = 205
CCB	9.8%
ACEI	9.8%
ARB	35.1%
Beta-blocker	12.2%
Nitrate	0.5%
Amiodarone	29.3%
Pacemaker	2.9%
Atrial fibrillation at rest	1.5%
CI to the use of atropine	2.4%

SD: standard deviation; CCB: calcium-channel blocker; ACEI: angiotensinconverting enzyme inhibitor, ARB: angiotensin II receptor blocker; CI: contraindication

**Table 2:** Risk factors for atherosclerotic disease

Risk factor	n= 205
SAH	64.3%
DM	12.7%
Smoking	7.8%
Dyslipidemia	33.2%
Previous AMI	6.3 %
MR	5.9%
FH	11.7%

SAH: systemic arterial hypertension; DM: diabetes mellitus; AMI: acute myocardial infarction; MR: previous myocardial revascularization; FH: family history of atherosclerotic disease.

Regarding the echocardiographic parameters and vital signs (Table 3), most patients had preserved ejection fraction, normal systolic and diastolic blood pressure, but heart rate tending to the lower limit of normality.

**Table 3:** Echocardiographic characteristics, blood pressure and heart rate

Variable	n	Mean	SD	95%CI	Median	IQR	Min	Max
LA (mm)	205	36.02	5.402	(35.28; 36.77)	36	(32; 40)	25	52
VST (mm)	205	8.698	1.504	(8.49; 8.905)	8	(8; 9)	5	14
PW (mm)	205	8.517	1.363	(8.329; 8.705)	8	(8; 9)	4	14
LVEDD (mm)	205	49.81	7.388	(48.79; 50.83)	50	(45; 54)	29	75
LVESD (mm)	171	30.13	5.566	(29.29; 30.97)	29	(26; 34)	20	61
EF (%)	205	62.36	11.16	(60.82; 63.89)	63	(58; 70)	28	88
SCIr*	205	1.23	0.362	(1.18; 1.279)	1.06	(1; .29)	1	2.47
SCIp*	205	1.249	0.415	(1.192; 1.306)	1	(1; 1.29)	1	2.8
SBP (mm Hg)	205	123.8	18.74	(121.2; 126.3)	120	(110;140)	80	180
DBP* (mm Hg)	205	74.54	9.518	(73.23; 75.85)	80	(70; 80)	60	110
HR (beat/min)	205	67.88	12.43	(66.17; 69.59)	66	(59; 75)	45	103

* Significant: variables without normal distribution. SD: standard deviation; 95%CI: 95% confidence interval; IQR: interquartile range; LA: anteroposterior measure of left atrium; VST: ventricular septal thickness; PW: posterior wall thickness; LVEDD: left ventricular end-diastolic diameter; LVESD: left ventricular end-systolic diameter; EF: ejection fraction; SCIr: segmental contractility index at rest; SCIp: segmental contractility index at peak; SBP: systolic blood pressure; DBP: diastolic blood pressure; HR: heart rate at rest.

More than half of the group (105 patients - 51.2%) had some alteration in segmental contractility at rest: in the apical segments of the ventricle, 30 patients; in the basal segments of the inferior and/or inferolateral wall, 35; association of the two alterations described, 32; and diffuse hypokinesia, 8. Regarding electrocardiographic changes, 98 patients had the following tracing alterations at rest: isolated right bundle branch block, 60 patients; association of right bundle branch block with left anterior hemiblock, 27; left bundle branch block, 5; atrial fibrillation rhythm, 3; and pacemaker rhythm, 3. In addition, only segmental contractility alteration, electrocardiographic alteration, or association of both was present in 50, 43 and 55 patients, respectively.

Negative result for myocardial ischemia was the most frequent finding in 139 exams (67.9%). That result was positive in 29 exams (14.1%), and inconclusive (did not reach submaximal heart rate) in 37 (18%). Of the patients with inconclusive result, 22 (59.5%) used maximum dose of dobutamine and underwent all stages of the protocol, but some had their exams interrupted because of the following: severe chest pain, 1 (2.7%); important blood pressure elevation (> 230/120 mm Hg), 1 (2.7%); severe headache, 2 (5.4%); and cardiac arrhythmias, 11 (29.7%). Frequent and polymorphic ventricular extrasystoles and non-sustained ventricular tachycardia were the most common arrhythmias related to exam interruption. Most patients with frequent ventricular extrasystoles during the exam had isolated extrasystoles at rest. Likewise, most arrhythmias were dose-dependent, occurring at pharmacological stress peak. Of the patients receiving maximum dose of dobutamine, 16 (72.7%) were on negative chronotropic drugs, such as beta-blocker or amiodarone.

Table 4 shows the arrhythmias induced during DSE. Of the 205 patients, 18 (8.7%) had more than one type of arrhythmia during the exam.

**Table 4:** Arrhythmias induced during stress echocardiography

Arrhythmias	n (%)
AF	1 (0.5%)
SSVT	2 (1%)
VE	100 (48%)
Bigeminismo	9 (4.4%)
NSVT	15 (7.3%)
SVT	2 (1%)

AF: atrial fibrillation; SSVT: sustained supraventricular tachycardia; VE: ventricular extrasystole; NSVT: non-sustained ventricular tachycardia; SVT: sustained ventricular tachycardia.

The protocol was interrupted because of the appearance of significant arrhythmias (atrial fibrillation, sustained supraventricular tachycardia, non-sustained ventricular tachycardia and sustained ventricular tachycardia). Those patients required neither specific drug nor electrical cardioversion. No patient had hemodynamic instability. All patients underwent routine observation.

Headache was the most frequent unwanted symptom (2.4%) during the exam, followed by chest pain (2.0%). No patient had hypotension during the exam, and only one (0.5%) had a hypertensive response.

No patient had significant adverse events, such as acute myocardial infarction, ventricular fibrillation, asystole, stroke, cardiac rupture or death.

## Discussion

The use of DSE to diagnose CAD in patients who cannot undergo exercise test has increased. In addition, more aggressive protocols with high doses of dobutamine and atropine have been more often used.^20^

To our knowledge, this is the first study designed to assess the safety of DSE, as well as the occurrence of arrhythmias, in an exclusive population of patients with Chagas disease.

Despite the potential risk of complications, mainly arrhythmogenic ones, the method was safe when applied to 205 patients. None had significant complications, such as death, acute myocardial infarction, cardiac rupture, stroke, ventricular fibrillation or asystole. Most safety studies have reported a very low incidence of those events: the meta-analysis by Geleijnse et al.,^18^ with 55,071 patients, has found an incidence of death, cardiac rupture and stroke lower than 0.01%, of acute myocardial infarction of 0.02%, and a rate of major complications of 1:475 (adding sustained ventricular tachycardia, asystole and ventricular fibrillation). Those figures are in accordance with those reported in the International Stress Echo Complication Registry,^21^ with a rate of 1:595 in the assessment of 35,103 patients.

The population studied belongs to the same age group of those of the studies on DSE safety assessed in the meta-analysis cited.^20^ Recently, a study conducted by O'Driskll et al.^22^ with 550 octogenarian patients has demonstrated that DSE was safe in that population and capable of identifying individuals at high risk for cardiovascular event.

The patients with Chagas disease had a lower prevalence of risk factors for CAD as compared to those without Chagas disease, in previous studies.^20,23,24^ Of those risk factors, the most prevalent were hypertension and dyslipidemia, the only ones that got closer to those of the non-chagasic populations studied, such as the group assessed by San Roman et al.^25^, with the following prevalence: hypertension, 61%; diabetes mellitus, 29%; dyslipidemia, 46%; smoking, 23%; history of previous infarction, 23%; and revascularization, 31%.

Regarding pharmacological treatment, Chagas disease patients used less frequently antianginal therapy, such as beta-blockers, nitrates and calcium-channel blockers, as compared to those of previous studies.^20,25^ However, 30% of the patients used amiodarone, an antiarrhythmic and negatively chronotropic drug, which might have accounted for not reaching submaximal heart rate in most inconclusive results.

In our study, the positive result for ischemia was less frequent than in other studies, maybe because of the smaller number of risk factors for CAD in the group of patients with Chagas disease.^20,23,24^ A cohort of 4,033 patients conducted by Mathias et al.^26^ has shown a positive result in 37% of them, and inconclusive result in 10%. Sicari et al.,^27^ in a cohort of 7,333 patients, has reported a positive result for ischemia in 39% of the exams.

The only study published, assessing Chagas disease patients submitted to DSE, has been conducted by Aquatella et al.^28^ That study aimed at assessing whether the stimulation with dobutamine could trigger an abnormal contractility response, as seen in ischemic myocardium. In that small cohort (24 Chagas disease patients vs 10 controls), dobutamine has shown a chronotropic incompetence and a reduced contractile response, even in those without apparent cardiac manifestation. That study might explain part of the inconclusive results found in ours, because of that probable chronotropic deficit.

Most patients with Chagas disease studied had some degree of segmental impairment, frequent in that pathology.^29^ The segmental contractility index, which reflects the segmental myocardial impairment extent, was slightly altered (median value, 1.06), reflecting mild alterations and few impaired segments.

Regarding arrhythmias, ventricular extrasystoles were the most frequently found, similarly to that reported in the safety studies analyzed in the meta-analysis by Geleijnse et al.^18^ However, the incidence was higher than that reported in most studies, that by Takeuchi et al. ^30^ being the one that got closer. That study, with 1,090 patients, has assessed different dobutamine-atropine protocols, with a 43.6% incidence of ventricular extrasystoles.^30^ Non-sustained ventricular tachycardia had the second highest incidence, 15 patients (7.3%), which was also greater than those already published, with a mean of 2.19% (range, 0.2% to 7.3%).^18^ The study conducted by Bremer et al.,^31^ with 4,035 patients, assessing the safety of stress echocardiography performed by nurses, was the only to show an incidence similar to the one of that group. Sustained ventricular tachycardia occurred in 2 (1%) patients, and that incidence was also higher than the one reported in previous studies for patients without Chagas disease, whose mean was 0.15% (range, 0.0% to 0.78%). Regarding supraventricular arrhythmias, the incidence was similar to that of other studies, where atrial fibrillation had a mean incidence of 0.9%, and sustained supraventricular tachycardia, of 1.3%.^18^ Our patients had 0.5% and 1.0%, respectively.

Unwanted adverse effects, such as chest pain, had a lower incidence than in previous studies, such as that by Mathias et al.,^32^ San Roman et al.^25^ and Mertes et al.,^20^ where chest pain occurred in 12.6%, 8.5% and 12.7%, respectively. Headache had the same frequency of that in other studies, as demonstrated by Mathias et al.,^32^ Mertes et al.^20^ and San Roman et al.,^25^ with incidence of 1.9%, 4% and 1.9%, respectively.

In addition, the incidence of hypertensive response and hypotension was lower than that of the safety studies assessed in the meta-analysis by Geleijnse et al.,^18^ in which the mean incidence of hypertension as the cause of protocol interruption was 1.3%, and that of hypotension, 1.7%. A recent retrospective analysis by Abram et al.,^33^ with 2,968 patients with no cardiovascular disease and normal findings on stress echocardiography, has shown that blood pressure variation during the exam depends on age, sex and use of atropine. A greater increase in systolic blood pressure was seen in men and young individuals, with a more pronounced effect of atropine among the young.

### Study limitations

This study is a retrospective analysis of a database, with the limitations inherent in that type of analysis. However, the exams were systematically performed by the same trained medical and nurse team, with large experience in that type of exam.

The database is small as compared to those of safety studies of stress echocardiography, but the identification of that type of patient is limited.

We had no coronary angiography of the patients who reported previous history of acute myocardial infarction. Thus, one might argue whether the segmental contractility alteration of such patients, when present, could be attributed to acute myocardial infarction or to Chagas heart disease. However, the number of those patients in our sample was reduced.

The interobserver variation analysis of the echocardiographic data could not be performed, because the digital images were not stored.

## Conclusions

Stress echocardiography with dobutamine and atropine showed to be safe in the population of patients with Chagas disease, in which no life-threatening outcome was observed.

The incidence of arrhythmias during the exam was higher than that found in studies with populations without Chagas disease.

The incidence of adverse effects, such as chest pain, arterial hypertension and hypotension, was lower than that found in studies with populations without Chagas disease.
